# How can we escape the ESKAPEs: Antimicrobial resistance mechanisms and what lies ahead?

**DOI:** 10.1371/journal.ppat.1012270

**Published:** 2024-06-13

**Authors:** Jessica B. Kelly, Aaron C. Nolan, Merve S. Zeden

**Affiliations:** Microbiology, Infectious Disease Section, School of Biological and Chemical Sciences, College of Science and Engineering, University of Galway, Galway, Ireland; University of Geneva: Universite de Geneve, SWITZERLAND

## Introduction

The ESKAPE pathogens are a group of 6 nosocomial pathogens: *Enterococcus faecium*, *Staphylococcus aureus*, *Klebsiella pneumoniae*, *Acinetobacter baumannii*, *Pseudomonas aeruginosa*, and *Enterobacter* spp., and are so-called, due to their ability to “escape” from our current arsenal of antimicrobials via a variety of mechanisms, leading them to be multidrug resistant (MDR) [1]. ESKAPE pathogens can possess the ability to exchange plasmid-encoded resistance genes by horizontal gene transfer, which leads to the continual rise of antimicrobial resistance (AMR) in these pathogens. The ESKAPE pathogens are in the critical or high-priority pathogen lists according to the World Health Organisation (WHO) [2] and are listed as urgent or serious threats to public health by the Centre for Disease Control and Prevention (CDC) [3]. Unfortunately, if nothing changes, we will be facing the consequences of a “post-antibiotic era,” with staggering effect on increasing global healthcare costs and worsening clinical outcomes [4].

Despite the differences between species (gram-positive and gram-negative) within the ESKAPE group, some of the overall strategies of resistance employed are conserved between them. This article summarises the intrinsic and acquired antimicrobial resistance mechanisms of the ESKAPE pathogens and discusses the future therapeutic avenues that are being explored in our global fight against AMR.

## What are the main AMR mechanisms employed by the ESKAPE pathogens?

Mechanisms of AMR can be categorised as intrinsic or acquired. Intrinsic resistance mechanisms are those that naturally occur within organisms, coded by genes in their chromosome [5]. Acquired mechanisms are the result of mutations in the bacterial chromosome following exposure to selective pressure, or by the acquisition of new genetic elements (i.e., mobile genetic elements (MGEs); plasmids, transposons, integrative elements) that can be incorporated into the bacterial genome following horizontal gene transfer [6].

**Fig 1:**
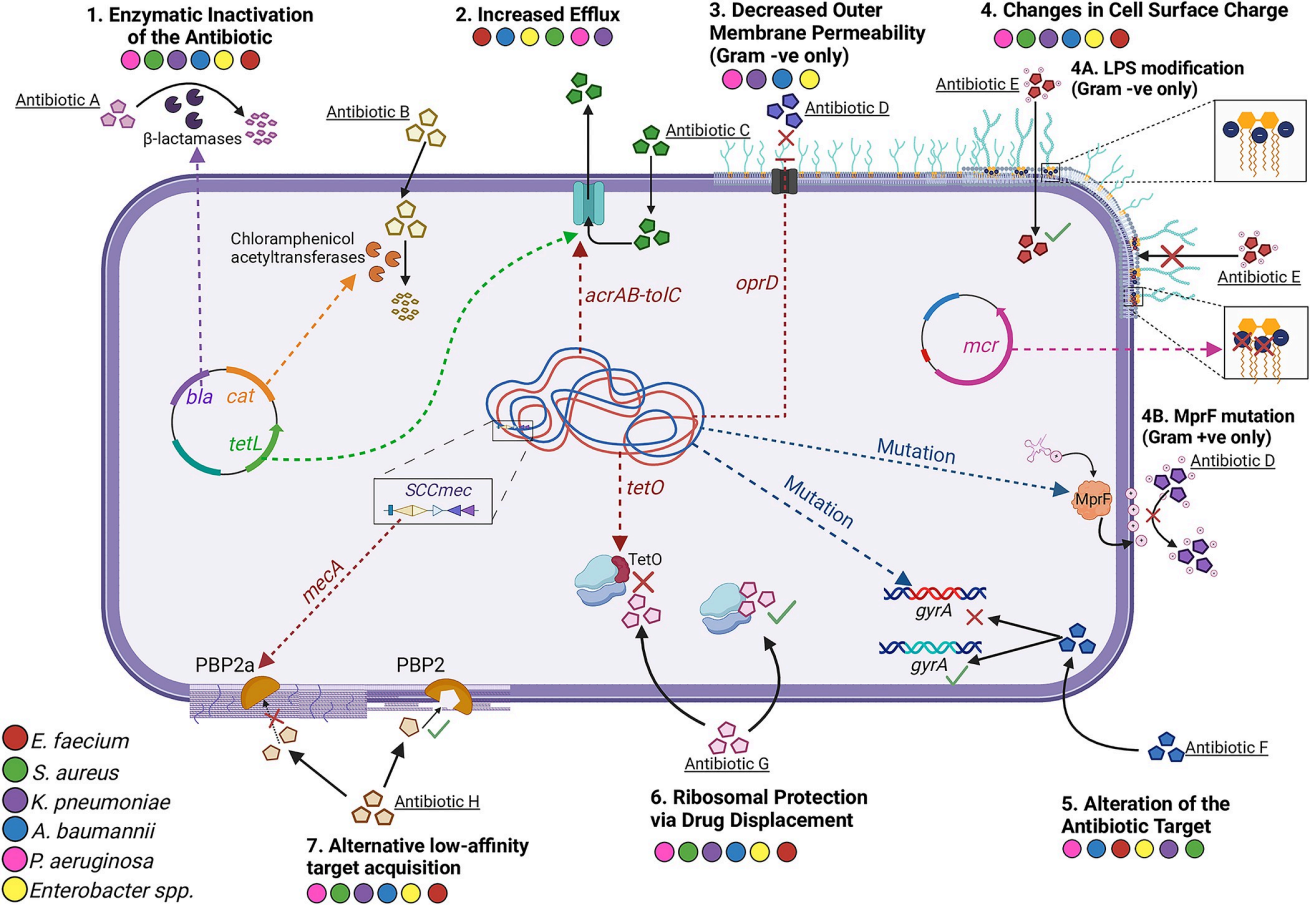
Schematic representation of 7 common resistance mechanisms that can be employed by individual strains of the ESKAPE pathogens. Each species is represented on the figure with a coloured spot; red = *E*. *faecium*, green = *S*. *aureus*, purple = *K*. *pneumoniae*, blue = *A*. *baumannii*, pink = *P*. *aeruginosa*, and yellow = *Enterobacter* spp. Schematic made using Biorender.com.

Here, we summarise 7 common ways ESKAPE pathogens can become resistant to antibiotics (Fig 1). It is important to note that within any given ESKAPE pathogen species not all strains utilise the mechanisms described.

1. **The enzymatic inactivation of an antibiotic** (Fig 1) is a mechanism that can be found in individual strains of all ESKAPE pathogens. Examples include β-lactamases and chloramphenicol acetyltransferases (CATs). β-lactamase enzymes, which are the most common mechanism of resistance to β-Lactams in gram-negative bacteria are secreted outside the cell and hydrolyse the β-lactam ring rendering the antibiotic ineffective [7]. CATs transfer an acetyl group from acetyl-Co-A to the 3-hydroxyl group of chloramphenicol, thus rendering the antibiotic unable to engage with its target, the 50S subunit of the bacterial ribosome [8].
2. **Using efflux pumps to extrude antibiotics from the cell** (Fig 1) prevents antibiotics from having detrimental impact on bacteria. There are 5 main classes of efflux pumps that have the ability to remove antibiotics from inside the cell where the antimicrobial target is located [9]. The resistance nodulation division superfamily is the most prevalent in gram-negative bacteria. MDR efflux pumps are present in both gram-positive and gram-negative species [1] and can be encoded on the chromosome or on plasmids (i.e., AcrAB-TolC, TetA, TetL, MexCD-OprJ, OqxAB) [10–14]. Chromosomally encoded efflux pumps often have quite a broad range of substrates thus they are important features of MDR species [1,9]. Additionally, bacteria can use horizontally acquired drug-specific pumps (i.e., TetA, TetL) to extrude specific antibiotics from the cells [12,14].
3. **Decreasing the permeability of the outer membrane** (Fig 1) is frequently utilised among gram-negative species. This reduces the amount of antibiotic that can enter the cell. A common way of doing this is through the loss of porin proteins, such as OprD in *P*. *aeruginosa*, preventing the antibiotic from entering the cell, hence blocking its activity, and causing resistance [15].
4. **Changes in cell surface charge** (Fig 1). Gram-negative bacteria can acquire plasmids encoding mobilised colistin resistance (*mcr*) genes, which confer resistance by reducing the overall negative charge of lipopolysaccharides (LPS) (Fig 1 and 4A). Bacteria can also accumulate intrinsic mutations affecting the overall expression of genes responsible for production of positively charged 4-amino-4-deoxy-L-arabinose and phosphoethanolamine, which are added to lipid A increasing its net charge (Fig 1 and 4A) [16]. The role of *mcr* genes in resistance to colistin and other polymyxin drugs, which are considered last resort antibiotics for gram-negative MDR infections, highlights the importance of this resistance mechanism [17]. Mutations in the multiple peptide resistance factor gene, *mprF* in *S*. *aureus* were found to confer cross resistance to last resort antibiotics vancomycin and daptomycin. Mutations in *mprF* lead to increased lysinylation of phosphatidylglycerol (Lys-PG), which increases the overall positive charge of gram-positive cells (Fig 1 and 4B). This leads to increased repulsion of positively charged daptomycin and decreased accessibility of vancomycin to its preferred cell wall substrate (Fig 1 and 4B) [18].
5. **Alteration of antibiotic targets** (Fig 1) involves acquisition of genetic mutations or phenotypic modifications that change important domains/motifs of proteins targeted by antibiotics. For example, mutations in *parC* and *gyrA* genes that encode DNA topoisomerase IV and DNA gyrase, the targets of many fluoroquinolones, prevent antibiotic binding and confer resistance [19]. Sometimes 1 or 2 base pair substitutions are sufficient to confer resistance. On the other hand, phenotypic alterations to the antibiotic targets that can mediate resistance to macrolide-lincosamide-streptogramin B (MLSB) antibiotics can be exemplified with the acquisition of chromosomal *ermA* or plasmid encoded *ermC*, which lead to methylation of ribosomal RNA (rRNA), thus conferring resistance to erythromycin [20–22].
6. **Ribosomal protection by drug displacement** (Fig 1) from the target in ESKAPE pathogens is exemplified by ribosome protection proteins (i.e., TetM, TetO) that can confer resistance to tetracycline by binding to the 70S ribosome leading to conformational change, GTP hydrolysis, and direct or indirect release of the antibiotic [23].
7. **Alternative low-affinity target acquisition** (Fig 1). Penicillin-binding proteins (PBPs) are enzymes involved in cell wall synthesis and are required to maintain the integrity and structure of the bacterial cell [24,25]. They are the target of β-lactam antibiotics which bind to PBPs and prevent them from carrying out their function, leading to weakening of the cell wall and cell death. Bacteria can acquire new PBPs with reduced affinity for β-lactams (i.e., PBP2a in *S*. *aureus*) (Fig 1, Mechanism 7), through the acquisition of the MGE Staphylococcal Cassette Chromosome *mec* (SCC*mec*). Accumulation of mutations in the genes encoding PBPs that reduce the β-lactam binding affinity is another commonly utilised mechanism in bacteria [24] (Fig 1, Mechanism 5). Acquisition of plasmid-encoded genes such as *vanA*, that encodes the vancomycin/teicoplanin A-type resistance protein, can lead to vancomycin resistance in the gram-positive ESKAPE pathogens. Typically, vancomycin binds to the d-alanine-d-alanine (d-ala-d-ala) dipeptide terminus of the peptidoglycan precursor lipid II in the gram-positive cell walls and inhibits the synthesis of peptidoglycan. When *vanA* is present, the target d-ala-d-ala is replaced by d-ala-d-lactate depsipeptide, which reduces the binding affinity of vancomycin, thus conferring resistance [26].

As discussed, ESKAPE pathogens have multiple resistance mechanisms (Fig 1) that they can utilise to overcome treatment with antibiotics, which has led to the current crisis of AMR. Furthermore, there are many ways bacteria can persist or tolerate antibiotics [27]. Understanding resistance, persistence and tolerance mechanisms is crucial for the development of new innovative approaches to improve treatment of MDR infections.

## What is the outlook for therapeutics against ESKAPE pathogens?

Since the approval of daptomycin (a lipopeptide) in 2003 [28], there have not been any new classes of antibiotics approved to treat the emerging threat of ESKAPE pathogens. It is imperative that novel therapeutic approaches are researched, so that we can keep pace with the rising levels of resistance to antibiotics in ESKAPE pathogens. At present, there are several interesting therapeutics in development, including anti-virulence molecules, photodynamic light therapy, bacteriophage therapy, anti-sense oligonucleotide (ASO) therapeutics, antimicrobial peptides (AMPs), vaccines, combination therapies, and repurposed drugs, to name a few [29,30]. Three of these novel therapeutics in development, namely bacteriophages, ASOs, and AMPs will be discussed in more detail below as the focus of this section.

**Bacteriophages** are naturally occurring viruses of bacteria that can be isolated from the environment and despite being listed here as novel therapeutics; phage activity has been researched for therapeutics since the 1920s. There are many qualities which make phages an attractive therapeutic, e.g., their ability to break up biofilm, their limited disruption to the commensal microbiota, and of course their ability to kill MDR bacteria [31]. Despite these advantages, there have also been several limitations to large-scale implementation of phage therapy, with the main one being the ability of bacteria to gain resistance to phage infection at a rapid rate. In addition to this, phages are also highly selective for their host bacteria, thus, for therapeutic purposes, the disease-causing strain must be identified. However, researchers are consistently discovering new strategies to overcome these limitations, an example of this is deploying phage cocktails instead of monophage therapy [32]. By including multiple phages for different bacterial targets in this alternative approach, researchers aim to target even the phage-resistant strains. Additionally, phage-antibiotic combination therapy has shown promising results in improving the clinical outcomes versus monotherapy [32,33]. Despite the limitations of phage therapy, it has been applied clinically in a number of circumstances, mainly in Russia and Georgia [34]. This is due to the continuation of bacteriophage research and development in these countries since the early 20th century. Despite success stories, phages have yet to become a standard therapeutic intervention in the fight against AMR [35,36]. This is partially owed to the current legislative framework, which does not provide much context for bacteriophage therapy [34]. However, at present several phage therapies are undergoing clinical trials in the west, which could lead to changes in the legislation, allowing phage therapy to become a standard therapeutic intervention in the future [31].

**Antisense oligonucleotides (ASOs)** refer to segments of nucleotide bases that could be paired with the coding region of target genes, causing the segment to be cleaved by RNase degradation, and therefore preventing their expression. This is a very targeted approach against bacterial survival, as it focuses only on specific genes of choice [37]. ASOs could be applied on their own or could be used in conjunction with antibiotic treatments to increase their efficacy against AMR pathogens [38]. Despite these advantages, this technology is still in its early phases, and there are many challenges to be overcome. For example, ASOs require modifications or attachments to facilitate their entry into cells due to the lack of conserved uptake mechanisms for nucleotides. With the advances in artificial intelligence (AI), continuous development in bioinformatic tools and databases, and advancements in research, some of the problems associated with ASO design and uptake may soon be overcome. ASO therapy has been shown to be effective in mouse models in several studies against numerous diseases and could become a fruitful technique as the technology and methodology improves [39].

**Antimicrobial peptides (AMPs)** are naturally occurring peptides expressed by a range of organisms including insects, plants, and humans that contribute to protection from pathogens [40]. AMPs can be deployed as therapeutic agents for bacterial infections due to their significant antibacterial properties [41]. AMPs are positively charged and interact with the negative charged membrane or LPS of the bacterial cell envelope causing membrane disruption [42] and cell death. Some AMPs can also enter the cell and interrupt important intracellular processes, e.g., DNA/RNA synthesis [42]. These are both examples of the direct activity of the AMPs on the bacterial cell, but they can also trigger a range of immunomodulatory effects increasing the rate that bacterial infection is cleared from the host [43]. Interest in AMPs as a novel drug category for treating infections caused by AMR bacteria is owed to the breadth of their mechanisms of action, which decreases the likelihood that resistance will develop in targeted pathogens [40]. Colistin and polymyxin B are 2 currently licenced AMPs with antibacterial properties but both have potentially serious side effects for patients [44] and are reserved for serious multidrug-resistant infections only. Limited *in vivo* efficacy and toxicity to host cells are barriers to the approval of new AMPs for clinical use. Current research to overcome these drawbacks, for example, with nanoparticle drug delivery, may improve the effectiveness of AMPs in the future [45,46].

Each of the examples discussed above are being researched as novel therapeutics on their own, and in combination with antibiotics. In the meantime, there is still a need for antibiotics to treat AMR infections. Despite the positive outlook for ASOs, bacteriophages, and AMPs, there is much research to be done before these are accepted as “gold standard” therapeutic agents.

At present, the use of several antibiotic combinations for extreme MDR infections caused by ESKAPE pathogens [47] is one example of how our current arsenal is being repurposed. However, limitations to this approach include severe side effects, antagonism with other drugs, and resistance to the antibiotic combinations. Indeed, since MDR bacteria have evolved in part due to the overuse and misuse of antibiotics, the increased use of antibiotics in combination therapy may lead to higher levels of resistance than monotherapy [48]. Therefore, it may be advisable to reserve the deployment of antibiotic combinations for only the most recalcitrant infections caused by ESKAPE pathogens.

## Conclusion

AMR is a serious global health threat that is predicted to cause 10 million deaths annually by 2050 without intervention [49]. Healthcare costs and mortality rates for patients infected with antibiotic resistant bacterial species are significantly higher than those infected with susceptible pathogens, highlighting the importance of the problem and the need for novel interventions [50–52]. Expansion of global research efforts, in combination with the implementation of antimicrobial stewardship, infection prevention and control measures, strict antibiotic prescription, and public education are essential to ensure a sustainable approach to this global problem [53]. This review highlights how emergence of AMR in bacteria is a natural and predictable outcome that necessitates the continuous development of novel, sustainable and innovative therapies as part of our global efforts in addressing the AMR crisis.
